# LEADERSHIP DEVELOPMENT TRAINING FOR BRAZILIAN ORTHOPEDIC SURGEONS

**DOI:** 10.1590/1413-785220243201e272375

**Published:** 2024-03-22

**Authors:** Verena Oberlohr, Vincenzo Giordano, José Octavio Soares Hungria, Marcelo Caiero, Robinson Esteves Pires, Luiz Henrique Penteado da Silva, Alexandre Pallottino, Gustavo Tadeu Sanchez, Pedro José Labronici, Madeline MacKechnie, Theodore Miclau

**Affiliations:** 1 University of California, Orthopaedic Trauma Institute, Department of Orthopaedic Surgery, San Francisco, School of Medicine, Zuckerberg San Francisco General Hospital, San Francisco, California, USA.; 2 Hospital Municipal Miguel Couto, Prof. Nova Monteiro Orthopedics and Traumatology Department, Rio de Janeiro, RJ, Brazil.; 3 Rede D’or São Luiz, Clínica São Vicente, Rio de Janeiro, RJ, Brazil.; 4 Santa Casa de Misericórdia de São Paulo, Orthopedic Trauma Group, São Paulo, SP, Brazil.; 5 Hospital das Clínicas da Faculdade de Medicina da USP, Institute of Orthopedics and Traumatology, São Paulo, SP, Brazil.; 6 Universidade Federal de Minas Gerais (UFMG), Department of the Locomotor System, Belo Horizonte, MG, Brazil.; 7 Instituto de Ortopedia e Traumatologia, Passo Fundo, RS, Brazil.; 8 Hospital Central Aristarcho Pessoa - CBMERJ, Orthopedics Department, Rio de Janeiro, RJ, Brazil.; 9 UNIFESP (Universidade Federal de São Paulo), Paulista School of Medicine, Department of Orthopedics and Traumatology, São Paulo, SP, Brazil.; 10 Hospital Santa Teresa, Prof. Donato D’Ângelo Orthopedics and Traumatology Department, Petrópolis, Rio de Janeiro, RJ, Brazil.

**Keywords:** Orthopedic Surgeons, Program Development, Latin America, Lower-Middle-Income Countries, Cirurgiões Ortopédicos, Desenvolvimento de Programas, América Latina, Países de Baixa e Média Renda

## Abstract

**Objective:**

To report on the experience and impressions of the Brazilian orthopedic trauma surgeons attending the Leadership Development Program (LDP) hosted by the Sociedade Brasileira do Trauma Ortopédico (SBTO) in Sao Paulo, Brazil on November 4, 2022.

**Methods:**

Forty-eight orthopedic trauma surgeons from five different regions throughout Brazil were provided a link to complete The Big Five Test, a validated online personality assessment. The questionnaire was available in Portuguese and was intended to provide a background on individual personality traits and their influence on interpersonal interactions. The LDP integrated content from literature reviews specific to Latin America, established leadership programs from leading business schools, and various subject matter experts. Prior to the start of the LDP, participants received a pre-course survey evaluating demographic information, a needs assessment, and the prioritization of leadership topics utilizing a 5-point Likert-scale. Attendees participated in the one-day, interactive LDP focusing on the fundamental principles of leadership development, communication, personal development, emotional intelligence and negotiation. Following the LDP, a post-course evaluation was administered to determine the participants’ overall experience, and suggestions for LDP improvement.

**Results:**

Forty-one of the forty-eight course participants completed the pre-course evaluation, whereas forty-six of the forty-eight participants completed the post-course evaluations. Overwhelmingly, the lack of opportunity was most prevalently reported as the main obstacle to attending a leadership course, as cited by 56% of respondents.

**Conclusion:**

Expanding the accessibility, diversity, and customizability of leadership programs can facilitate the development of personal tools needed to move healthcare forward. Critical topics include emotional intelligence and other differentiating leadership qualities that distinguish true transformational and servant leaders. Advancing leadership skills can stimulate networking, expose learners to experiential learning styles, inspire others to create positive change, and engender creative solutions for systematic improvements and health outcomes. ***Level of Evidence III; Individual Case-Control Studies.***

## RESUMO

**Objetivo:**

Relatar a experiência e as impressões de cirurgiões de trauma ortopédico brasileiros participantes do Programa de Desenvolvimento de Liderança (PDL), organizado pela Sociedade Brasileira do Trauma Ortopédico (SBTO), em São Paulo, Brasil, em 4 de novembro de 2022.

**Métodos:**

Quarenta e oito cirurgiões de trauma ortopédico de cinco regiões diferentes do Brasil receberam um link para preencher o The Big Five Test, uma avaliação de personalidade on-line validada. O questionário estava disponível em português e pretendia fornecer informações básicas sobre traços de personalidade individuais e sua influência nas interações interpessoais. O PDL integrou conteúdo de análises de literatura específicas da América Latina, e programas de liderança estabelecidos pelas principais escolas de negócios e por vários especialistas no assunto. Antes do início do PDL, os participantes receberam uma pesquisa pré-curso solicitando informações demográficas, uma avaliação de necessidades e a priorização de tópicos de liderança utilizando uma escala Likert de 5 pontos. Os participantes participaram do PDL interativo de um dia com foco nos princípios fundamentais de desenvolvimento de liderança, comunicação, desenvolvimento pessoal, inteligência emocional e negociação. Após o PDL, foi realizada uma avaliação pós-curso para determinar a experiência geral dos participantes e sugestões para melhoria do PDL.

**Resultados:**

Quarenta e um dos quarenta e oito participantes do curso concluíram a avaliação pré-curso, enquanto quarenta e seis dos quarenta e oito participantes concluíram a avaliação pós-curso. A falta de oportunidade foi relatada com maior prevalência como o principal obstáculo para frequentar um curso de liderança, conforme citado por 56% dos entrevistados.

**Conclusão:**

Expandir a acessibilidade, a diversidade e a personalização dos programas de liderança podem facilitar o desenvolvimento de ferramentas pessoais necessárias para fazer avançar os cuidados de saúde. Os tópicos críticos incluem inteligência emocional e outras qualidades de liderança diferenciadas, que distinguem verdadeiros líderes transformacionais e servidores O avanço das competências de liderança pode estimular o networking, expor os alunos a estilos de aprendizagem experiencial, inspirar outros a criar mudanças positivas e gerar soluções criativas para melhorias sistemáticas dos resultados na saúde. ***Nível de Evidência III; Estudos de caso-controle individuais.***

## INTRODUCTION

There is increasing value in the awareness and enhancement of cognitive and social skillsets. Such cognitive skills include decision-making, planning, and situational awareness, while the social component encompasses leadership, teamwork, and communication skills.^1^ In the general clinical setting, it has been shown that these skillsets are highly associated with the capacity of a physician to practice medicine according to standards of intellectual and moral excellence, bearing the responsibility for patient care, medical education and research, and cultural organization.^2^ In the context of orthopedic trauma, these non-technical skills have profound implications on patient outcomes and the performance of healthcare systems.^3–7^ Human behaviors relating to these skillsets have been implicated in nearly 50-80% of errors or adverse events in medicine.^7,8^ This reinforces that these abilities are fundamental to clinical expertise and the delivery of high-quality,^8^ safe, effective, and patient-centered care.^9^

While the assessment and training of these non-technical skills have become more accessible in high-income countries (HICs), there remains a need for further development and incorporation of these tools into the medical education throughout low- and middle-income countries (LMICs),^1^ particularly throughout Latin America.^10^ Given the direct association between national income and spending, a significant portion of the care delivered in LMICs is often greatly hindered by the lack of requisite healthcare funding. The burden of disease is greater, advanced technology is limited, the supply of human and material resources is inconsistent, and the healthcare systems remain less integrated than in many HICs. As a result, the individual and collective disharmony, dissatisfaction, and disappointment often experienced in the doctor-patient relationship is commonly attributed to this adverse environment.^11^ The effect that human behavior may have on patient safety and health outcomes may thus be more substantial in LMICs than in HICs.^1^ Despite their classification as upper middle-income countries, Brazil and other Latin American countries are characterized by unequal wealth distribution, thereby having significant proportions of their populations to conditions that are more commonly found in countries with a lower gross domestic products.^12^ These healthcare challenges are further in situations where non-technical skillsets assist in improved management of the available resources, prompting further interest in understanding and improving these skills.

In 2019, a cross-sectional, multinational survey was administered to the Asociación de Cirujanos Traumatólogos de las Americas (ACTUAR) network, an international collaborative consortium established to enhance research capacity among orthopedic trauma surgeons in Latin America.^12^ The survey was designed to determine the interest in and relative importance of various leadership topics and other non-technical skills. The survey was completed by 144 orthopedic surgeons from 18 Latin American countries.^10^ The results characterized region-specific perspectives, desired competencies, and existing barriers to leadership development participation and formed the basis for a novel Leadership Development Program (LDP) curriculum for Latin American orthopedic trauma surgeons.^10^

Through this collaborative effort, an inaugural LDP was actualized in 2019 in Hermosillo, Mexico; followed by Havana, Cuba; Veracruz, Mexico; and most recently, Sao Paulo, Brazil. This paper will report on the experience and impressions of the Brazilian orthopedic trauma surgeons attending the LDP hosted by the Sociedade Brasileira do Trauma Ortopédico (SBTO) in Sao Paulo, Brazil on November 4, 2022.

## MATERIALS AND METHODS

Forty-eight orthopedic trauma surgeons from five different regions throughout Brazil were invited to attend a 1-day LDP on the basis of their experience and/or leadership responsibilities. The course was attended by 92.7% males and 7.3% females, having an average of 10 years of practice experience since training. All participants were board certified members of the Sociedade Brasileira de Ortopedia e Traumatologia (SBOT) and active members of the SBTO. According to the official division of Brazilian regions, 10.5% lived in the south, 87.5% in the southeast, and 2% in the northeast of the country.

In preparation for the course, participants were provided a link to complete The Big Five Test,^13^ a validated online personality assessment. The questionnaire was available in Portuguese and was intended to provide a background on individual personality traits and their influence on interpersonal interactions. Prior to the start of the course, participants received a link to complete a pre-course evaluation to determine their general interest and experience in leadership development opportunities as well as their prioritization of various leadership topics.^14–16^ Upon course completion, a post-course evaluation was administered to assess course efficacy, obtain suggestions for improvement, and capture the overall experience of attendees.

The LDP integrated content from literature reviews specific to Latin America, established leadership programs from leading business schools, and various subject matter experts.^13^ In addition to illustrating the fundamental principles of leadership development, the curriculum cultivated various social and cognitive skillsets, including personal learning styles, communication, team dynamics, personal development, emotional intelligence, strength deployment inventory, and negotiation. To accommodate diverse learning styles, these concepts were presented using hands-on learning activities, case studies, real-world applications, interactive group activities, and formal didactic instruction. The course content and course materials were primarily provided in English.

The 2022 board of the SBTO fully supported the course and was actively present throughout its administration. The original study was deemed “exempt” by UCSF and given the study number 19-28517.

## RESULTS

### Pre-Course Evaluation

Forty-one (85%) of the forty-eight course participants completed the pre-course evaluation. Respondents reported a current leadership position (97.6%), most commonly within the hospital setting (92.7%), with an equal distribution among different ranges of leadership experience (0-2 years, 3-5 years, and 6+ years). Despite 100% of respondents expressing interest in attending a leadership course, only 14.6% reported previous leadership course attendance. Overwhelmingly, the lack of opportunity was most prevalently reported as the main obstacle to attending a leadership course, as cited by 56% of respondents. (Table 1)

**Table 1:** Pre-Course Evaluation Results: Leadership Position and Leadership Course Attendance (Reported as % of Respondents).

Currently in Leadership Position	Leadership Setting	Years of Experience in Leadership Position	Previous Leadership Course Attendance	Barriers to Leadership Course Participation
97.7%	Clinic – 29.3%	0-2 years – 31.7%	14.6%	Limited Opportunities – 56%
	Hospital – 92.7%	3-5 years – 29.3%		Early in Career – 29.3%
	Regional Society – 14.6%	6+ years – 36.6%		Schedule Conflicts – 12.2%
	National Society – 9.6%			Cost – 4.9%
	International Society – 0%			Work Schedule Conflicts – 17%
				Other – 2.4%

Source: Sociedade Brasileira do Trauma Ortopédico and University of California at San Francisco, 2022.

**Figure 1:**
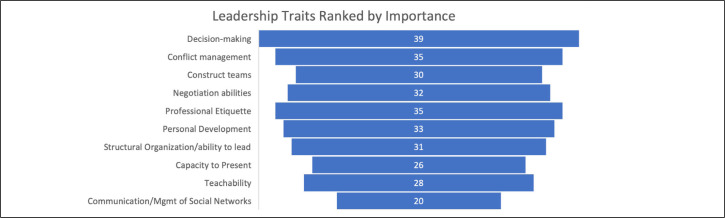
Leadership Traits Ranked

Respondents were asked to evaluate the most important leadership topics utilizing a 5-point Likert scale, assigning items a rank between 1- indicating “strongly agree” and 5- indicating “strongly disagree”. Decision-making ability, professional etiquette, and conflict management were ranked among the most important topics (Figure 1). Notably, when respondents were asked to propose additional leadership topics, a common thread emerged around introspective development, which included continued personal and professional growth, adaptability, self-improvement, open mindedness, and support for emerging leaders. Many of these commonly identified leadership qualities were universally acknowledged social and cognitive skills and well represented throughout the LDP content. When asked to preferentially rank various learning styles, lecture-based and simulation exercises were rated most favorably. (Table 2)

**Table 2:** Pre-Course Evaluation Results: Learning Style Preferences (Reported as Frequency Cited).

Learning Style	Preference
Lecture	26
Simulation Exercises	21
Interactive Plenary Session/Panel Discussion	15
Small Group Work	18

Source: Sociedade Brasileira do Trauma Ortopédico and University of California at San Francisco, 2022.

### Post-Course Evaluation

Post-course evaluations were completed by forty-six (96%) of the forty-eight participants, 96% of whom attended the full duration of the course. Respondents unanimously agreed that the course material was enthusiastically communicated, that it had clearly articulated goals, and that the course design was conducive to achieving these goals. Furthermore, 100% of respondents concurred that the course encouraged active participation through discussions and group activities. Commonly cited course strengths included the novel and dynamic content as well as the interactive exercises. Suggestions for improvement included providing pre-course and other written materials for added background, expanding and referencing the various topics covered, and providing simulation and group activity instructions in Portuguese.

Collectively, these course evaluations demonstrate that orthopaedic trauma surgeons have a variety of leadership responsibilities. These evaluations also showed that there was a perceived paucity of leadership development opportunities available to Brazilian orthopedic trauma surgeons, confirming an interest in pursuing such opportunities.

## DISCUSSION

The role of an orthopedic trauma surgeon requires specialization, expertise, and the assumption of various responsibilities that require intrinsic leadership qualities. Properly developed leadership knowledge and skills are pivotal to protecting the patient’s interests, organizational direction and requirements, and professional integrity.^2–17^ Leadership development has been identified as one of the most important priorities for medical education this century;^18^ however, the inclusion of this subject in medical training curricula remains inconsistent and lacks standardization across countries.^18,19^ According to Brazil’s national curriculum guidelines for medical education, leadership skills are considered a component of the basic knowledge a physician must have to work in interdisciplinary teams with responsibility, empathy, effective communication, decision making, and the assumption of leadership positions.^20,21^

Nevertheless, most medical schools don’t formally integrate these expected these subjects into the curriculum.^20^ Historically, surgeons haven’t been trained to focus on leadership or reflect on their personal behavioral style.^22^ By the nature of their profession, they tend to focus on outcomes rather than the processes involved in achieving those outcomes.^23^ Consequently, physician leaders are often selected on the basis of their success in the core activities of medical centers: research, education, and patient care; yet they may often lack sufficient training and experience in administration, management, and leadership.^24^ Only by understanding this longstanding cultural reality, medical doctors will be able to develop an expansive new framework for prioritizing societal health care needs and expectations, instead of exclusively focusing on the individual patient.^25^

Furthermore, there are limited studies reporting on leadership education in Brazil or evaluating the efficacy of leadership integration in medical practices.^19^ A systematic review evaluating leadership development programs for U.S.-based physicians demonstrated that physician leadership has focused on imparting technical and conceptual knowledge, customarily through lectures and seminars.^25^ These teaching tools often take precedence over efforts to build self-awareness, for which action-based learning, feedback, and self-development activities may be more appropriate.^25^ Notably, studies documenting favorable organizational outcomes were characterized by the use of multiple learning methods, including lectures, seminars, group work, and action learning projects in multidisciplinary teams.^23–26^ Incorporating diverse learning formats, adapting for the personality types of different learners, and teaching concepts over time are more likely to become part of their daily behavior such that they become second nature.^21^ Moreover, an extended program duration can cultivate valuable networking opportunities, as participants who spend significant periods of time learning together often develop a special camaraderie, which encourages ongoing collaboration and synergy among colleagues and institutions for the encouragement of leadership behavior.^21^ As collaboration continues to progress, participants must adapt and embrace roles of leadership.^2^

As teamwork and collaboration are increasingly fundamental to healthcare operations, there is a growing need to include self-awareness and emotional intelligence as fundamental competencies within LDPs.^24^ The most successful emerging healthcare models will effectively address the shift from individualism to a culture of collaboration and interaction – a transition largely driven by emotional intelligence.^26^ From the boardroom and chairman’s office to the ward and bedside, emotional intelligence is ubiquitous throughout clinical settings^25^ and represents an element of leadership that ultimately influences individual and collective efforts to accomplish shared objectives.^27^ Emotional intelligence and its concomitant skills are valued non-technical abilities within the personal development toolkit and have been identified as the most essential competency for leaders to succeed in academic institutions and other organizations.^28^ To this end, two essential components of emotional intelligence must be present in a leader; social skill, or the talent to propagate others in a desired direction; and empathy, the sensitivity to feelings and emotions of others.^29^ The introspective leadership qualities proposed by SBTO leadership course participants in the pre-course evaluations reflect a high sensitivity and capacity for prioritizing and further developing these abilities. Cultivating leadership skills to empower self-aware, empathetic, altruistic, and authentic leaders who demonstrate commitment to the growth of people and communities can have profound systemic implications on healthcare.^21^ Leadership skill education has been an eclectic activity, recently moving away from a hierarchical and exclusively empirical process, towards the lived experience of the leader.^30^

## CONCLUSION

Expanding the accessibility, diversity, and customizability of leadership programs can facilitate the development of personal tools needed to move healthcare forward. Critical topics include emotional intelligence and other differentiating leadership qualities that distinguish true transformational and servant leaders. Advancing leadership skills can stimulate networking, expose learners to experiential learning styles, inspire others to create positive change, and engender creative solutions for systematic improvements and health outcomes.
